# High-Throughput Screen Detects Calcium Signaling Dysfunction in Hutchinson-Gilford Progeria Syndrome

**DOI:** 10.3390/ijms22147327

**Published:** 2021-07-07

**Authors:** Juan A. Fafián-Labora, Miriam Morente-López, Fco. Javier de Toro, María C. Arufe

**Affiliations:** Grupo de Terapia Celular y Medicina Regenerativa, Departamento de Fisioterapia, Ciencias Biomédicas y Medicina, Universdidade da Coruña, Agrupación Estratégica INIBIC-CICA, 15006 A Coruña, Spain; juan.labora@udc.es (J.A.F.-L.); miriam.morente.lopez@udc.es (M.M.-L.); javier.toro@udc.es (F.J.d.T.)

**Keywords:** HGPS, Ca^2+^ signaling, GRP75, GRP78, NAC, ROS

## Abstract

Hutchinson–Gilford progeria syndrome (HGPS) is a deadly childhood disorder, which is considered a very rare disease. It is caused by an autosomal dominant mutation on the LMNA gene, and it is characterized by accelerated aging. Human cell lines from HGPS patients and healthy parental controls were studied in parallel using next-generation sequencing (NGS) to unravel new non-previously altered molecular pathways. Nine hundred and eleven transcripts were differentially expressed when comparing healthy versus HGPS cell lines from a total of 21,872 transcripts; ITPR1, ITPR3, CACNA2D1, and CAMK2N1 stood out among them due to their links with calcium signaling, and these were validated by Western blot analysis. It was observed that the basal concentration of intracellular Ca^2+^ was statistically higher in HGPS cell lines compared to healthy ones. The relationship between genes involved in Ca^2+^ signaling and mitochondria-associated membranes (MAM) was demonstrated through cytosolic calcium handling by means of an automated fluorescent plate reading system (FlexStation 3, Molecular Devices), and apoptosis and mitochondrial ROS production were examined by means of flow cytometry analysis. Altogether, our data suggest that the Ca^2+^ signaling pathway is altered in HGPS at least in part due to the overproduction of reactive oxygen species (ROS). Our results unravel a new therapeutic window for the treatment of this rare disease and open new strategies to study pathologies involving both accelerated and healthy aging.

## 1. Introduction

Hutchinson–Gilford progeria syndrome (HGPS) is a deadly childhood genetic disorder caused by an autosomal dominant mutation on the LMNA gene and is a very rare disease [1]. HGPS belongs to a group of diseases called laminopathies, all of them sharing mutations in the LMNA gene as the causal agent. The LMNA gene encodes for lamins A and C and these have important structural and regulatory functions in the nuclear envelope [2]. HGPS is characterized by premature aging of the organism and accelerated senescence at the cellular level [3], and patients die at a mean age of 13 years old, mainly due to cardiovascular complications. The LMNA point mutation most frequently associated with HGPS promotes the processing and accumulation of a deleterious lamin A isoform, called progerin or lamin A Δ50 [4], which remains permanently farnesylated [5]. In recent years, therapeutic strategies have focused on reverting the action of progerin, mainly through the use of farnesyltransferase inhibitors [6]. Almost a decade ago, Viteri et al. demonstrated that progerin promotes the accumulation of oxidized proteins in fibroblast cell lines derived from HGPS patients [7]. More recently our group demonstrated that the deregulation of lamin A alters the oxidative stress balance in mesenchymal stem cells (MSCs), modifying their migratory properties and inducing defects in their capacity to differentiate into chondrocytes in our in vitro model. Furthermore, our group has reported that progerin expression induces mitochondrial dysfunction and ROS overproduction [8]. These changes, at least in part, increase protein folding and autophagic proteolysis, indicating an overall loss of proteostasis that would lead to the process of premature aging [9]. The endoplasmic reticulum (ER) is an intracellular organelle essential for protein synthesis, folding, and modification, and it also maintains calcium homeostasis within the cell. Cellular function and survival are potentially regulated by Ca entry through store-operated Ca entry (SOCE) channels. These are essential for maintaining intracellular Ca stores [10]. The Ca release from ER stores is controlled by the inositol 1,4,5-trisphosphate receptors (IP3R). Cell survival depends on the ability of cells to respond to ER stress since chronic stress leads to apoptosis [11]. Mitochondria respond to cellular stress by releasing proteins, for example, cytochrome C from the space between their membranes, controlling the intrinsic pathway. Cellular stress is manifested as heat, infection, hypoxia, or calcium and nutrient deprivation. These deregulations are associated with the development of diseases such as cancer and tissue damage after increased apoptosis. Apoptosis is the highly controlled process of programmed cell death used by multicellular organisms to clean damaged cells and to maintain cell numbers in the functional organs [12]. The membrane contact site between mitochondria and the endoplasmic reticulum is known as mitochondria-associated membrane (MAM) [13]. This contact site is crucial for various biological events, such as lipid transfer, sterol exchange, Ca transfer, apoptosis, and the suppression of neurological disorders [14]. Previously, we have identified several calcium signaling-related proteins as being significantly modulated in an in vitro model of progerin over-expression [9]. Chen et al. [15] have demonstrated the involvement of ROS and/or altered Ca signaling in HGPS. Our goal in this study was to deepen the understanding of the alteration in calcium signaling in premature aging in human cells from HGPS patients, focusing on the contribution of MAM to the underlying mechanism.

## 2. Results

### 2.1. RNAseq Analysis of HGPS and Healthy Control Cell Lines

**Figure 1:**
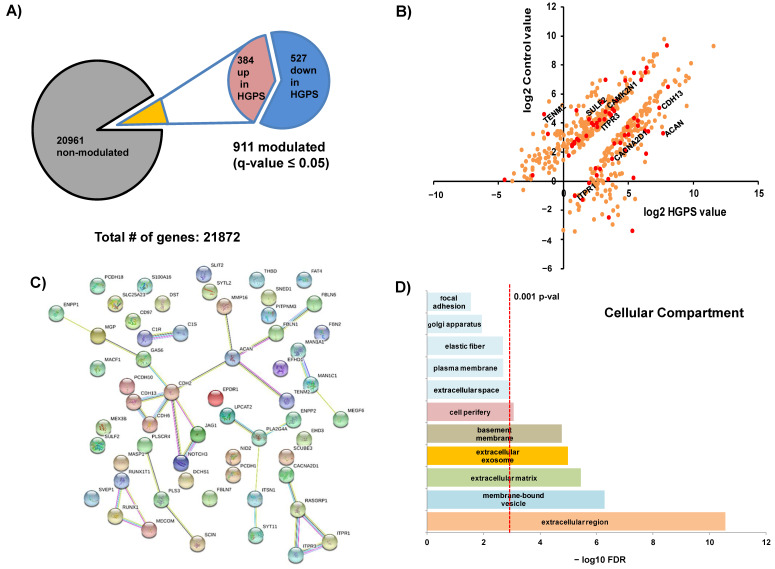
Summary of the massive genomic analysis (RNAseq). (**A**) Summary of the NGS showing the 21,872 different quantified transcripts. Of these, 911 were considered to be significantly modulated, with *q*-values ≤ 0.05. (**B**) Representation of the values for the 911 significantly modulated transcripts and those coding for Ca^2+^ flux-related proteins (in red). (**C**) String 10.1 pathway analysis for the Ca^2+^ flux-related transcripts showing the interaction networks. (**D**) Statistical analysis shows that most of those transcripts codify for extracellular and membrane-bound (vesicle/exosome) proteins.

Genomic datasets were deposited in the Gene Expression Omnibus (GEO-NCBI) repository (http://www.ncbi.nlm.nih.gov/geo/, accessed on 30 April 2018). The raw files are public and freely accessible (www.ncbi.nlm.nih.gov/geo/query/acc.cgi?acc=GSE113648, accessed on 30 April 2018). This is an analysis of a previously published at PLoS ONE [16] data set carried out by our group. From a total of 21,872 transcripts measured, 911 were detected as differentially expressed when comparing healthy versus HGPS cell lines with a q-value less than 0.05. A summary of the NGS showing the quantified transcripts is presented in Figure 1A. The list of significantly modulated transcripts was imported into String 10.1 software for pathway analysis (Figure 1B–D). Transcripts involved in Ca^2+^ signaling are indicated in Figure 1B and Table A1. The proteins associated with those transcripts were used for the network (Figure 1C) and cellular component analysis (Figure 1D), indicating that most of the proteins are located in membranes and extracellular regions.

### 2.2. Orthogonal Validation of Ca^2+^ Flux-Related Proteins and Transcripts

**Figure 2:**
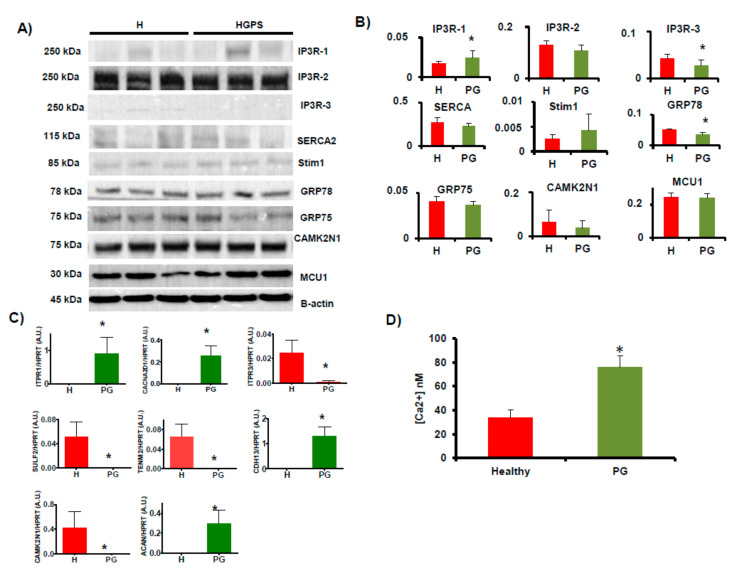
Study of receptors involved in Ca^2+^ signaling. (**A**) Western blots of lysates from healthy (AG03257, AG03258, AG03512, and AG06299) and HGPS (AG03198, AG03199, AG03513, and AG06917) human skin fibroblasts. Antibodies used are indicated in Table A2. (**B**) Densitometry for normalized proteins relative to β-actin. (**C**) Histograms representing different gene expression levels normalized with HPRT used as housekeeping gene in healthy and HGPS human skin fibroblasts. (**D**) Total basal Ca^2+^ measured in healthy (AG03257, AG03258, AG03512, and AG06299) and HGPS (AG03198, AG03199, AG03513, and AG06917) human skin fibroblasts using a luminescence spectrometer. Bars are means ± SEM from three independent experiments. * *p* < 0.05 was considered statistically significant. H = healthy, PG = HGPS.

Orthogonal validation of NGS results was performed using a battery of Ca^2+^ transporters localized in MAM (Table A2 and Table A3). Western blot analysis demonstrated that IP_3_R1 was significantly (*p* < 0.05) higher in HGPS vs. healthy cell lines and IP_3_R3 was significantly (*p* < 0.05) lower in HGPS vs. healthy cell lines. In addition, the level of GRP78 was statistically significantly lower in HGPS than in healthy cell lines (Figure 2A,B). The qRT-PCR analysis demonstrated that *ITPR1*, *CACNA2D1*, *CDH13*, and *ACAN* expression were significantly (*p* < 0.05) higher in HGPS vs. healthy cell lines and ITPR3, CAMK2N1, SULF2, and TENM2 expression were significantly (*p* < 0.05) lower in HGPS vs. healthy cell lines. All of these results validated the results obtained by shotgun analysis (Figure 2C).

### 2.3. Calcium Dynamics Measurement

**Figure 3:**
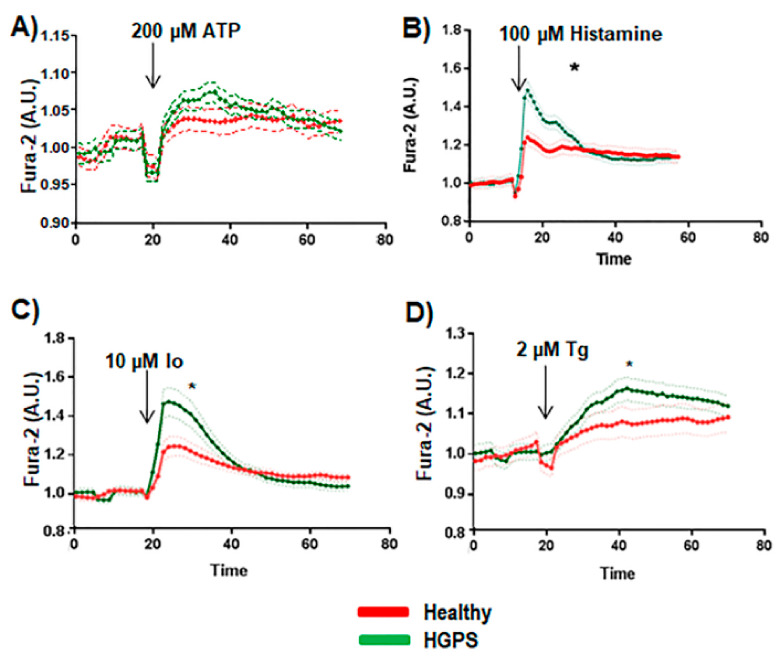
Cytosolic calcium handling study. (**A**) Ca^2+^ flux in the absence of extracellular calcium with 3 mM of EGTA in the medium was performed using healthy (AG03257, AG03258, AG03512, and AG06299) and HGPS (AG03198, AG03199, AG03513, and AG06917) human skin fibroblasts in a FlexStation reader. (**B**) Results after adding ATP at 100 μM (arrow). (**C**) Results after adding 10 μM ionomycin (Io) (arrow). (**D**) Results after adding 10 μM thapsigargin (Tg) (arrow). The average calcium baseline levels were 33.61 ± 6.5 nM for WT cells and 76.21 ± 9.09 nM for HGPS cells. Bars are means ± SEM from three independent experiments. * *p* < 0.001 was considered statistically significant.

Basal calcium measurement by means of luminescence spectrometry revealed that HGPS cell lines had a statistically significant basal calcium level that was higher than that of the healthy cell lines (Figure 2D). The release of Ca^2+^ from HGPS cell lines and the healthy ones was not affected by ATP in a statistically significant way (*p* < 0.05) (Figure 3A). However, a statistically significant (*p* < 0.05) increase in the Ca^2+^ release was observed in HGPS vs. control cell lines when 100 µM histamine, 10 µM Io, or 2 µM Tg were added to the medium (Figure 3B–D). These results indicate that the proteins involved in the transport of Ca^2+^ are affected in HGPS.

### 2.4. Effect of ROS Scavengers in the Ca^2+^ Flux

**Figure 4:**
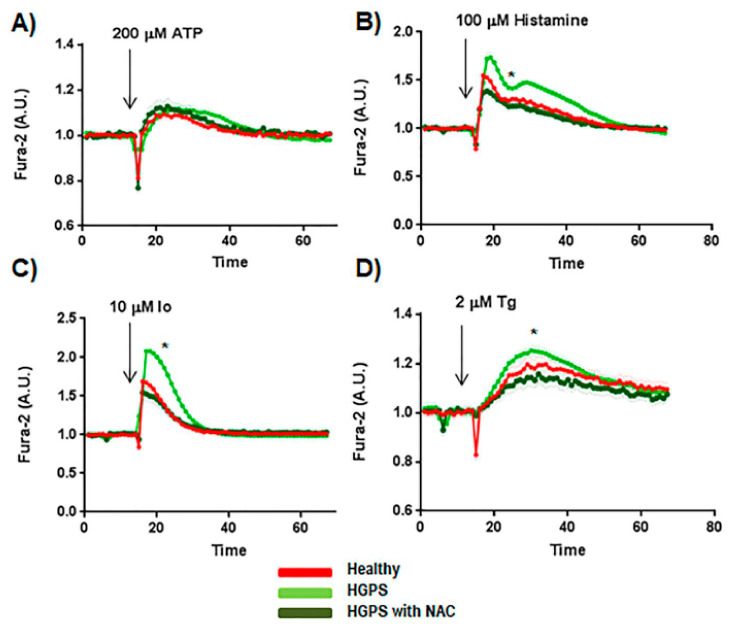
Cytosolic calcium handling study using NAC. (**A**) Ca^2+^ flux in the absence of extracellular calcium with 3 mM of EGTA in the medium was performed using healthy (AG03257, AG03258, AG03512, and AG06299) and HGPS (AG03198, AG03199, AG03513, and AG06917) human skin fibroblasts in a FlexStation reader. Some experiments were performed by adding NAC at 10 μM for one hour in the medium with HGPS cell line plates. (**B**) Results after adding ATP at 100 μM (arrow). (**C**) Results after adding 10 μM ionomycin (Io) (arrow). (**D**) Results after adding 10 μM thapsigargin (Tg) (arrow). Bars are means ± SEM from three independent experiments. * *p* < 0.001 was considered statistically significant.

**Figure 5:**
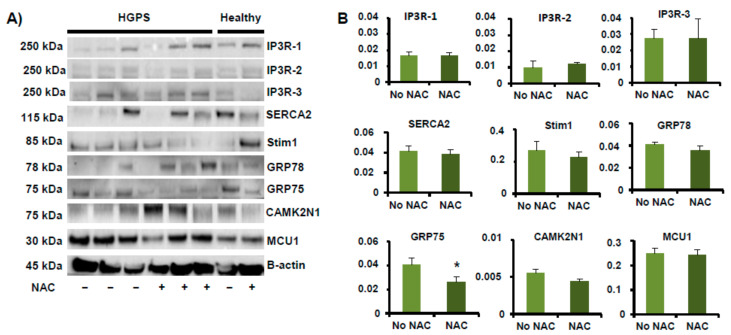
Effect of NAC on receptors involved in Ca^2+^ signaling. (**A**) Western blots of lysates from HGPS (AG03198, AG03199, AG03513, and AG06917) human skin fibroblasts incubated with 10 μM NAC and without treatment. The antibodies used are indicated in Table A2. (**B**) Densitometry results for normalized proteins relative to β-actin. Bars are means ± SEM from three independent experiments. * *p* < 0.05 was considered statistically significant.

FlexStation results for HGPS cell lines pre-treated for 1 h with 10 µM NAC are shown in Figure 4. These results show luminescence levels similar to the healthy cell lines when 100 µM histamine, 10 µM Io, or 2 µM Tg were added to the medium (Figure 4B–D). After treatment with NAC, the HGPS lines showed a statistically significant (*p* < 0.05) decrease in the GRP75 level. However, no differences were found in the levels of the rest of the proteins located in the MAM and involved in cytosolic calcium handling (Figure 5A,B).

**Figure 6:**
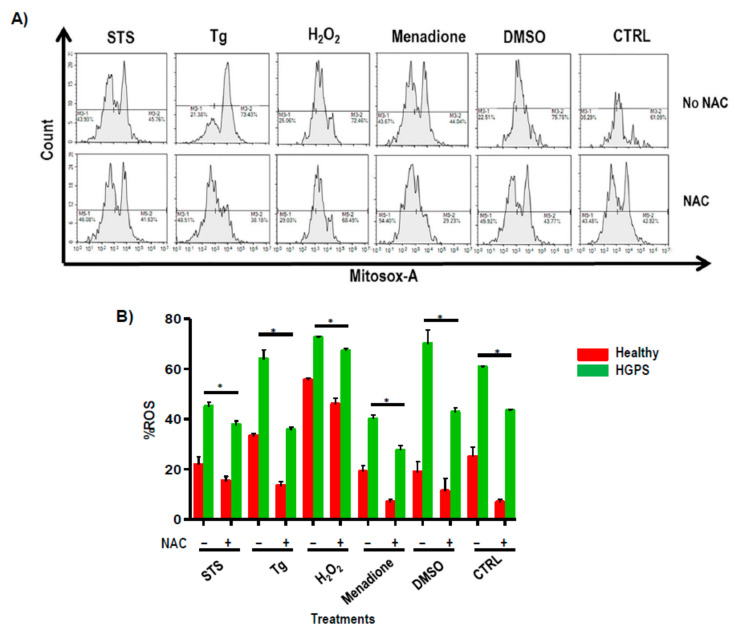
Reactive oxidative species study. ROS percentage assessed by means of flow cytometry from healthy (AG03257, AG03258, AG03512, and AG06299) and HGPS (AG03198, AG03199, AG03513, and AG06917) human skin fibroblasts treated with or without NAC. (**A**) Representative FAC histogram of mitochondrial superoxide activity using NovoExpress software (Agilent, CA, USA) from HGPS skin fibroblasts treated with or without NAC. (**B**) Histograms represent percentages of ROS. Bars are means ± SEM from three independent experiments. * *p* < 0.05 was considered statistically significant versus healthy cell lines. STS = staurosporine; Tg = thapsigargin; H_2_O_2_ = hydrogen peroxide; DMSO = dimethyl sulfoxide.

Cytometric results indicated that cells treated with H_2_O_2_ were the most ROS-producing (75 ± 2%), followed by cells treated with Tg (70 ± 3%). The addition of NAC to the medium decreased the production of ROS statistically significantly (*p* < 0.05) in all cell types, regardless of the treatment used on them (Figure 6A,B). STS is an inducer of apoptosis due to the elevation of intracellular free calcium levels and the accumulation of mitochondrial ROS. Menadione can induce the release of calcium from mitochondria. These results indicate that an excess of ROS in the system contributes to defects in the transportation of Ca^2+^ in HGPS.

### 2.5. Apoptosis Study

**Figure 7:**
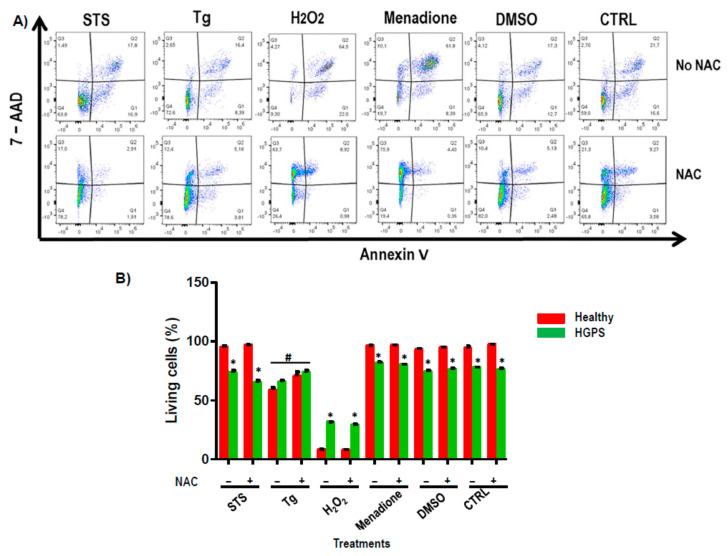
Apoptosis study. Flow cytometry from healthy (AG03257, AG03258, AG03512, and AG06299) and HGPS (AG03198, AG03199, AG03513, and AG06917) human skin fibroblasts treated with and without NAC. (**A**) Representative FAC profile showing the 7-ADD in front of Annexin V staining using FlowJo software V8.6 (Treestar, OR, USA) from HGPS skin fibroblasts treated with or without NAC. (**B**) Histograms represent the percentage of living cells with the different treatments. Bars are means ± SEM from three independent experiments. * *p* < 0.05 was considered statistically significant versus healthy cell lines. # *p* < 0.05 was considered statistically significant versus treatments. STS = staurosporine; Tg = thapsigargin; H_2_O_2_ = hydrogen peroxide; DMSO = dimethyl sulfoxide.

The results of flow cytometry analysis indicated that HGPS cell lines were significantly (*p* < 0.05) more sensitive to apoptosis when treatments that affected Ca^2+^ pathways were added to the medium (65 ± 3%) compared to healthy cell lines (95 ± 2%), except with Tg. Surprisingly, H_2_O_2_ treatment produced a statistically significant increase in apoptosis in healthy cell lines versus HGPS (Figure 7A). Statistically significant differences were found in the HGPS lines treated with Tg that had been pre-treated with NAC, indicated by an increase in the percentage of living cells (Figure 7A,B).

## 3. Discussion

In the last decade, the exponential development of massive genomics techniques has allowed the study of modulated molecular pathways in health and disease. In the present work, NGS technology permitted us to discover new pathways affected by the overexpression of progerin. One of the identified pathways is that of Ca^2+^ signaling, which is of paramount importance for the metabolic processes of organisms. We observed the upregulation of IP3R1 and the downregulation of IP3R3 in HGPS cells, which was orthogonally validated by Western blot analysis. These results suggest that HGPS is a complex metabolic syndrome, as pointed by other authors [17] and, consequently, it must be treated accordingly. The most affected receptors located in MAM and involved in Ca^2+^ signaling were IP3R1 and IP3R3, determined by Western blot analysis, both of which are essential receptors of Ca^2+^ and essential molecules for life. Upregulation of IP3R1 was responsible for the increased Ca^2+^ leak and the lowered [Ca^2+^] ER in the in vitro model described by Kasri et al. [18]. Similar results were published by Lo et al. [19] using iPS-ECs from HGPS patients to study transient receptor potential (TRP) channels.

Glucose regulatory protein 78 (GRP78), a master ER chaperone, is present at the MAM, where it folds steroidogenic acute regulatory protein (StAR) for delivery to the outer mitochondrial membrane. Thus, GRP78 is an acute regulator of steroidogenesis at the MAM, regulating the intermediate folding of StAR, which is crucial for its activity [20]. According to Walker et al. [21], a specific anti-tumor treatment increased intracellular Ca^2+^ levels, increased reactive oxygen species (ROS) levels and reduced GRP78 expression in a Ca^2+^-dependent manner. Mitochondrial Ca^2+^ uniporter’s pore (MCU) is required to allow the Ca^2+^-dependent activation of oxidative signaling [22]. However, our NGS study indicated that MCU was not affected in HGPS cell lines. The imbalance of IP3R1 and IP3R3, together with GRP78, found in HGPS cell lines could explain, at least partially, the high concentration of intracellular Ca^2+^ in HGPS cells.

To invoke a cytosolic Ca^2+^ release through IP3Rs in the ER, we applied ATP to activate metabotropic purinergic receptors in our cell lines. To induce a Ca^2+^ release largely from sensitive ER stores, we added Io or Tg, producing a slow liberation of more than 90% of the ER Ca^2+^ content in the Ca^2+^-free extracellular solution. Our results demonstrate that Ca^2+^ transport is compromised in HGPS cells since its release from intracellular organelles was increased in a statistically significant way when Io or Tg was added to the system. Our observations could be explained by a decrease in the GRP78 level, together with the imbalance of IP3R1 and IP3R3 levels found in the HGPS cell lines versus healthy ones. Furthermore, ROS production affects several functions localized at the MAM, which are involved in local Ca^2+^ transport [18].

The mitochondria-mediated route is an important apoptotic pathway in cells. To examine this route, NAC was added in our HGPS cell lines to alleviate the oxidative stress in mitochondria, and therefore in the MAM, and discern whether the ROS-scavenger agent could restore, at least partially, the normal intracellular Ca^2+^ flow in HGPS cell lines. GRP78 is an ER molecular chaperone that is a major UPR target with anti-apoptotic properties. GRP78 enhances the functional ability of the ER, promotes the survival and invasiveness of cancer cells, and confers chemoresistance [23]. It was observed that Ca^2+^ flow in healthy cell lines was similar to that in HGPS cells pre-incubated with NAC. To explain these results, immunoblotting was performed for the receptors involved in cytosolic calcium handling in MAM. Only the GRP75 level decreased in a statistically significant way in HGPS cells treated with NAC. Lv et al. indicated that GRP75-mediated ER-mitochondrial Ca^2+^ transfer upregulation of GRP75 produced an enhancement of reactive oxygen species (ROS) [24]. Therefore, NAC would downregulate the levels of GRP75 in the MAM, and this could contribute to the normalization of the Ca^2+^ flux observed in our study.

It is well known that HGPS cell lines show increased oxidative stress, which induces cell injury through mitochondrial-mediated cell apoptosis [19]. In a study focused on mitochondria, Baumgartner et al. [25] were the first authors who concluded that elevated Ca^2+^ was the crucial factor in causing cells to undergo oxidative-induced apoptosis. Our results indicated that the production of ROS was statistically significantly higher in HGPS compared to healthy cells independently of treatments, and pre-incubation with NAC produced a statistically significant decrease in ROS production in all the groups. This is in agreement with Kim et al. [26] and Richards SA et al. [27], who demonstrated that both ROS and Ca^2+^ signaling play roles in the disruption of mitochondrial homeostasis and the precedence of ROS production over the failure of Ca^2+^ flux homeostasis. Under physiological conditions, the Ca^2+^ transfer from the endoplasmic reticulum (ER) to mitochondria occurs at least in part at contact points between them in the MAM and involves the VDAC1/Grp75/IP3R1 complex. The ROS depletion from HGPS cells when NAC is added to the system could inhibit the GRP75 level, therefore decreasing protein interaction within the complex, attenuating the mitochondrial Ca^2+^ overload, and leading the normal flux of intracellular Ca^2+^. Paillard et al. [28] showed that a depression of GRP75 protected cardiomyocytes from the accumulation of Ca^2+^ in the mitochondrial matrix. The MAM is a complex matrix, composed of multiple proteins interacting between organelles to trigger the formation of functional channels that maintain cellular homeostasis.

We performed an apoptosis study in our system by pre-treating the cell lines with NAC to decrease the oxidative stress, and at the same time they were incubated with different treatments involving the Ca^2+^ pathway. STS was used to induce a cytotoxicity response by inhibiting the PI3K/AKT signaling pathway [29] and Tg was used to decrease respiration selectively, with complex I substrates producing an increase in ER stress, resulting in an intracellular calcium overload that favors the activation of calcium-dependent calpains, according to Mohsin et al. [30]. H_2_O_2_ was used to directly activate IP3 receptors [31], and reagents such as DMSO were used to induce ER stress, increasing Ca^2+^ levels through oxidative stress [32]. Menadione was used to induce the production of ROS, leading to oxidative stress and subsequent apoptosis. All the treatments used produced a significant decrease in HGPS cell survival compared with healthy cell lines and no differences were found between the HGPS and healthy cells pre-incubated with NAC except with Tg and H_2_O_2_.

Treatment with 2 µM Tg plus NAC produced a statistically significant increase in living cells. It is likely that NAC has a protective effect, inhibiting calcium depletion in the ER and consequently increasing cell survival [33]. Rivera-Torres et al. [31] indicated that defective cardiac repolarization and cardiomyocyte connectivity were important abnormalities in the pathogenesis of HGPS, increasing the risk of arrhythmia and premature death and their connection with Ca^2+^ flow and reducing sarcoplasmic reticulum calcium loading and release. Similarly, when applying Tg to HGPS cells, there was an increase in the surviving cells, which was higher when NAC was present in the system. These results indicate that oxidative damage causes a selective downregulation of the neuronal stress response, activated under conditions of ER dysfunction. Paschen et al. proposed that oxidative stress was implicated in mechanisms leading to neuronal cell injury in various pathological states of the brain using Tg [32].

Surprisingly, HGPS cells had a statistically significant higher survival than healthy ones when treated with H_2_O_2_, which could be explained by the fact that GRP78 and GRP75 are reduced in HGPS cell lines. The low glucose-induced apoptosis, targeting the endoplasmic reticulum and/or mitochondrial Ca^2+^, was explained by Younce et al. and Scrima et al. [33,34].

Our results indicated that the MAM could be involved in the imbalance of flow Ca^2+^ observed in our HGPS cell lines. However, the specific molecular mechanism to demonstrate that the basal intracellular accumulation of Ca^2+^ in HGPS is due to the imbalance of receptors from the MAM involved in Ca^2+^ signaling has to be establish through in vitro studies with HGPS mutant mice. The human HGPS cell lines from patients have a lot of genetic variability.

## 4. Materials and Methods

### 4.1. Cell Culture

Healthy (AG03257, AG03258, AG03512) and HGPS (AG03198, AG03199, AG03513) human skin fibroblasts, obtained from the National Institute of Aging (NIA) Aged Cell Repository (distributed by the Coriell Institute, Camden, NJ, USA), were maintained in DMEM, high glucose, GlutaMAX™ Supplement (Life Technologies, Merelbeke, Belgium) containing 10% fetal bovine serum (FBS) and supplemented with antibiotics. Experiments were performed within four passages following the arrival of the cells.

### 4.2. RNA-Seq Protocol

RNA was isolated using the TRIzol^®^ (Thermo Fisher Scientific, Madrid, Spain) extraction method. The quality of 1 µL of each RNA sample was checked using the Agilent Bioanalyzer 2100 to determine the RNA integrity number (RIN) score using the Agilent 6000 Nanochip and reagents (Agilent, St. Clara, CA, USA). Samples with a RIN score > 7 were retained and converted to cDNA using the SureSelect Strand Specific RNA library Prep for Illumina multiplexed sequencing method. One microgram of total RNA per sample was generated. The study was designed to screen the complete transcriptome sequence of normal (AG03257 and AG03512) and HGPS (AG03199 and AG03513) human skin fibroblasts. The sequencing data was generated on HiSeq 1500 on a rapid-mode flow cell from Illumina. Sample preparation and sequencing were conducted in duplicate. This procedure has been used and published previously by our group [16].

### 4.3. Bioinformatic Analysis

An average of 23 million paired-end 100-bp reads was obtained per sample. The raw RNA-Seq reads for each sample were aligned to the reference human genome browser (GRCh38.p12 assembly) using Bowtie2 (http://bowtie-bio.sourceforge.net/index.shtml, accessed on 12 November 2017) and Tophat2 (http://tophat.cbcb.umd.edu/, accessed on 10 February 2014. After alignment, raw sequence read depths were converted to estimate transcript abundance, measured as fragments per kilobase of exons per million (FPKM), and the Cufflinks (http://cufflinks.cbcb.umd.edu/, accessed on 5 May 2014) of differentially expressed genes and transcripts were calculated with Cuffdiff. Groups were compared. The fold-change thresholds had to be higher than 1.2 and lower than 0.8. Identified genes with statistically significant changes were categorized according to their function, biological processes, and cellular components using the R/Bioconductor package RamiGO (http://bioconductor.org/packages/release/bioc/html/RamiGO.html, accessed on 30 April 2018). This procedure has been used and published previously by our group [16].

### 4.4. qRT-PCR

The amplification program consisted of an initial denaturation at 92 °C for 2 min, followed by 40 cycles from 92 °C for 15 s, annealing at 55–62 °C, depending on the gene, for 30 s and extension at 72 °C for 15 s. PCR analysis was performed in triplicate, with each set of assays repeated three times. To minimize the effects of unequal quantities of starting RNA and to eliminate potential sources of inconsistency, relative expression levels of each gene were normalized to ribosomal protein (*HPRT*) using the 2^−ΔΔCt^ method [35]. The primers used for the amplification of human genes are described in detail in Table A2. Control experiments utilized no reverse transcriptase.

### 4.5. Western Blot Analysis

Proteins were obtained via the addition of RIPA buffer to obtain cell lysates from cell lines. Protein concentrations were determined using the BCA reagent (Bio-Rad Laboratories, Madrid, Spain) following the commercial protocol. Immunoblot analysis was performed on 40 μg of protein, which was resolved on NuPAGE 4–12% Bis-Tris gels (Invitrogen, Thermo Fisher Scientific, Madrid, Spain), and the blots were probed with antibodies directed against human antigens involved in Ca^2+^ signaling of MAM (Table A3).

### 4.6. Basal Calcium Measurement

A Luminescence Spectrometer (LS50B, Perkin Elmer, Zaventem, Belgium) was used for the basal calcium measurement of healthy and HGPS cell lines. In summary, de-esterification was carried out with Fura-2 (Thermo Fisher, Brussels, Belgium) (1/1000 *v*/*v*), consisting of 1 × 10^6^ cells in 2.5 mL of 1.5 mM Ca^2+^ Krebs glucose for 30 min at room temperature. Each cell line was measured at 340 nm excitation and 510 nm emission for 400 s. Digitonin was adding to check Rmax and EGTA to check Rmin. The formula used was:

(1) $$[{\mathrm{Ca}}^{2+}]=\frac{Kd\times \left(\frac{Sf2}{Sb2}\right)\times \left(R-Rmin\right)}{Rmax-R}$$

Kd = dissociation constant of Fura-2 for Ca^2+^ at room temperature (241 nM)
R = basal ratio
Rmin = minimum ratio (R-value after the addition of EGTA)
Rmax = maximum ratio (R-value after the addition of digitonin)
Sf2 = F380 max
Sb2 = FF380 min

### 4.7. Cytosolic Calcium Handling

The intracellular Ca^2+^ release in cultured primary skin fibroblasts from healthy and HGPS patients was measured. Ca^2+^-free extracellular medium supplemented with 3 mM EGTA was used to exclude Ca^2+^ entry across the cell membrane. Then, 10 × 10^3^ cells per well (96 wells/plate) were incubated with 2 μM Fura-2 for 30 min and washed twice with Ca^2+^ free standard external solution, including the following: 10 mM HEPES, 120 mM NaCl, 5.4 mM KCl, 1 mM MgCl2, 10 mM glucose, and pH 7.4 buffer. In some experiments, HGPS cell lines were pre-incubated with 10 μM of N-Acetyl-L-Cysteine (NAC) for one hour. The fluorescence intensity of fura-2-loaded cells was monitored using an automated fluorescent plate reading system (FlexStation 3, Molecular Device, Sunnyvale, CA, USA). A monochromator dual-wavelength-enabled alternative excitation was at 340 and 380 nm, whereas the emission fluorescence was monitored at 510 nm. SoftMax Pro Software (Molecular Devices, Sunnyvale, CA, USA) was used to process the images of multiple cells collected at each excitation wavelength and to provide ratios of fura-2 fluorescence (F340/F380). The baseline calcium levels were normalized for WT and HGPS cells using GraphPad Prism6 (GraphPad Software, La Jolla, CA, USA).

Cells were stimulated with Histamine at 100 μM in Ca^2+^-free conditions to mobilize Ca^2+^ from the stores, and ATP (200 μM final concentration) was applied to activate cell-surface purinergic receptors and induce subsequent IP3 production and Ca^2+^ release. Furthermore, 10 μM Ionomycin (Io) or 2 μM Thapsigargin (Tg) were applied to the cells to determine ER Ca^2+^ content.

### 4.8. Flow Cytometry

Flow cytometry was performed using Attune NxT (Thermo Fisher, Brussels, Belgium) equipment to study the apoptosis potential of HGPS cell lines and their healthy progenitors. Cells were washed twice in PBS (Sigma-Aldrich, St. Louis, MO, USA) and incubated with 1 μM staurosporine (STS), 1 μM Tg, 200 μM H_2_O_2_, 60 μM menadione, or 1 μM DMSO for 5 h prior to the apoptosis analysis. Incubation with FITC -7-AAD and PE-Cy5.5-annexin V was performed to check the potential apoptosis involved with mitochondrial membrane potential and oxidative stress. Then, 2 × 10^5^ cells were analyzed using FlowJo software V8.6 (Tree Star, San Carlos, CA, USA). Mitochondrial ROS was measured after the incubation of cells with 5 mM of Mitosox-A. The stained cells were then washed twice with PBS, and 2 × 10^5^ cells were analyzed using NovoExpress Software (Acea Biosciences, San Diego, CA, USA). For negative control staining, we used FITC-conjugated mouse IgG1K isotype, PE-conjugated mouse IgG1K isotype, PE-Cy5.5-conjugated mouse IgG1K isotype, and APC-conjugated mouse IgG1K isotype (all from BD Pharmingen, Madrid, Spain).

### 4.9. Statistical Analysis

All experiments were performed in duplicate, and one representative result is shown. Non-parametric statistical analysis was performed using the Mann–Whitney U and Kruskal–Wallis tests using GraphPad Prism6 (GraphPad Software, La Jolla, CA, USA). All results were presented as the standard error of the mean. *p* < 0.05 was considered statistically significant.

## 5. Conclusions

Our data show that HGPS, caused by an autosomal dominant mutation on the LMNA gene, produces a metabolic alteration, which could be associated with the MAM, destabilizing intracellular Ca^2+^ homeostasis. The normal Ca^2+^ flux can be partially rescued through the scavenging of ROS by NAC. In summary, HGPS involves the unbalanced homeostasis of the cellular inter-compartmental calcium flux and these new pieces of evidence point to ER and/or mitochondrial calcium transporters as targets of a new therapeutic strategy.

## Appendix A

**Table A1:** Transcripts involved in Ca^2+^ signaling.

Gene Name	Protein Name	RNAsec Data: Up in	Cellular Component
*ITPR1*	Inositol 1,4,5-trisphosphate receptor type 1	HGPS	Endoplasmic reticulum membrane
*CACNA2D1*	Voltage-dependent calcium channel subunit alpha-2/delta-1	HGPS	Sarcoplasmic reticulum, extracelular exosome, plasma membrane
*ITPR3*	Inositol 1,4,5-trisphosphate receptor type 3	Healthy	Endoplasmic reticulum membrane
*CAMK2N1*	Calcium/calmodulin-dependent protein kinase II inhibitor 1	Healthy	Endoplasmic reticulum membrane
*SULF2*	Extracellular sulfatase Sulf-2	Healthy	Golgi apparatus, endoplasmic reticulum
*TENM2*	Teneurin-2	Healthy	Nucleus, Golgi apparatus, endoplasmic reticulum, plasma membrane
*CDH13*	Cadherin-13	HGPS	Plasma membrane
*ACAN*	Aggrecan core protein	HGPS	Extracellular matrix

**Table A2:** Primers for real-time reverse transcriptase-polymerase chain reaction (qRT-PCR) amplification.

Gene Name	Forward 5′	Reverse 5′	mRNA ID
*ITPR1*	CCTGTTCAAGTTGTGTGCGA	CCTCCCACCAACTGAGATGT	NG_016144.1
*CACNA2D1*	AGTGGGAGGAGTGAGAGGAT	TCTACAATCCCAAACGGCCT	NG_009358.2
*ITPR3*	GTCTCCTGCTCCCTGTCTTT	AGAAATAAAGGTCGGGGCCA	NG_027729.1
*CAMK2N1*	TTGCAGTCTGTTGTGTGAGC	CCAAGCATTCCTGTACGTGG	NM_018584.6
*SULF2*	AAGGATGGTGGGGACTTCAG	CGGACTTCCCTCAGGTTCTT	AB033073.2
*TENM2*	CCTTTCAACAGCAGCTTGGT	CACCTCCATTTGCGATCAGG	AB032953.2
*CDH13*	GAATGAGAAAGCAGGCGAGG	TTGCCCTCCATGAGCTGTTA	NG_052819.1
*ACAN*	TCCCCGTAGAAGAGGAGACA	AAGACAGGGGTATGCAGCTT	NG_012794.1
*HPRT*	ATGACCAGTCAACAGGGGACA	GCTTGCGACCTTGACCATCT	NG_012329.2

**Table A3:** Ca ^2+^ transporters localized in MAM.

Gene Name	Antibody	Source	Commercial Brand
*ITPR3*	Inositol 1,4,5-trisphosphate receptor type 3	Rabbit	ACC-010 Alomone Labs.
*VDAC*	Voltage-dependent anion-selective channel protein 1	Rabbit	AVC-001 Alomone Labs.
*ITPR1*	Inositol 1,4,5-trisphosphate receptor type 1	Rabbit	ACC-019 Alomone Labs.
*GRP78*	Endoplasmic reticulum chaperone BiP	Rabbit	3183 Cell Signaling
*MCU*	Calcium uniporter protein, mitochondrial	Rabbit	ACC-328 Alomone Labs.
*SERCA2*	Sarcoplasmic/endoplasmic reticulum calcium ATPase 2	Rabbit	ACP-012 Alomone Labs.
*STIM1*	Stromal interaction molecule 1	Rabbit	ACC-063-AO. Alomone Labs.
*GRP75*	Stress-70 protein, mitochondrial	Rabbit	3177. Cell Signaling
*Tubulin*	Tubulin alpha-1B chain	Rabbit	2128S. Cell Signaling
*B-actin*	Actin-related protein 3B	Rabbit	4970S. Cell Signaling
*TOM20*	Mitochondrial import receptor subunit TOM20 homolog	Mouse	612278 BD
*CALM1*	Calmodulin-1	Mouse	Ab-38841. Abcam
	Anti-rabbit IgG-HRP	Goat	Sc-2004. Sta. Cruz
	Anti-mouse IgG-HRP	Goat	Sc-2005. Sta. Cruz
